# In Vivo Feasibility of Electrostatic Precipitation as an Adjunct to Pressurized Intraperitoneal Aerosol Chemotherapy (ePIPAC)

**DOI:** 10.1245/s10434-016-5108-4

**Published:** 2016-02-02

**Authors:** Tinatin Kakchekeeva, Cedric Demtröder, Nirmitha I. Herath, Dominic Griffiths, Jared Torkington, Wiebke Solaß, Marie Dutreix, Marc A. Reymond

**Affiliations:** 1 Department of Surgery, Otto-von-Guericke University Magdeburg, Magdeburg, Germany; 2 Department of Surgery, Marien Hospital Herne, Ruhr University Bochum, Herne, Germany; 3 DNA Therapeutics, Évry, France; 4 Alesi Surgical Ltd., Cardiff, Wales, UK; 5 Welsh Institute for Minimal Access Therapy (WIMAT), Cardiff, Wales, UK; 6 Institute of Pathology, Medical School Hanover, Hannover, Germany; 7 Institut Curie, Orsay, France

## Abstract

**Background:**

Intraperitoneal chemotherapy is limited by tissue penetration. Pressurized intraperitoneal aerosol chemotherapy (PIPAC) has been shown to improve drug uptake by utilizing the physical properties of gas and pressure. This study investigated the effect of adding electrostatic precipitation to further enhance the pharmacologic properties of this technique.

**Methods:**

A comparative study was performed using an in vivo porcine model. There were 3 cases in each group, PIPAC and electrostatic precipitation pressurized intraperitoneal aerosol chemotherapy (ePIPAC), plus 1 negative control comparing intraperitoneal distribution and tissue uptake of 2 tracer substances (toluidine blue and DT01). Tracer uptake was determined by measuring DT01 in tissue and peritoneal fluid at the end of each procedure.

**Results:**

Electrostatic precipitation of the aerosol was technically feasible in all ePIPAC animals. The aerosol was cleared completely from the visual field within 15 s in the ePIPAC group versus 30 min in the PIPAC group. The peritoneal surface was homogeneously stained in both groups. After 30 min, 1.5 % remaining DT01 was measured in samples of ePIPAC-treated peritoneal fluid versus 15 % in PIPAC animals (*p* = 0.01). Tissue concentration was increased after ePIPAC versus PIPAC (*p* = 0.06).

**Conclusions:**

ePIPAC is technically feasible and improves tissue uptake of 2 tracer substances compared to PIPAC by up to tenfold. Intraperitoneal distribution was homogeneous in both groups. ePIPAC has the potential to allow more efficient drug uptake, further dose reduction, a significant shortening of the time required for PIPAC application, and improved health and safety measures.

**Electronic supplementary material:**

The online version of this article (doi:10.1245/s10434-016-5108-4) contains supplementary material, which is available to authorized users.

Research on tumor nonresponse to therapy has focused on the molecular mechanisms of chemoresistance, with drug distribution being comparatively neglected.^1^ For cytostatic drugs to be successful, they must fully penetrate the tissue of interest, reaching within all the cancer cells at a concentration sufficient to exert a therapeutic effect.

Intraperitoneal tumor dissemination and metastasis is common in several forms of abdominal cancer.^2^ Numerous studies have investigated the potential role of intraperitoneal drug delivery as an adjunct to systemic chemotherapy in this situation.^3^ The rationale of intraperitoneal administration is to improve the therapeutic index by increasing the exposure of cancer cells within the peritoneal cavity to the drug while minimizing toxic effects to other organs. Prior studies have documented the limitations of intraperitoneal chemotherapy including the limited direct penetration of drugs into the tumor tissue and the unequal drug distribution throughout the peritoneal cavity.^4^

Pressurized intraperitoneal aerosol chemotherapy (PIPAC) is an innovative drug delivery system that takes advantage of the physical properties of the combination of gas and pressure in order to overcome these pharmacologic limitations.^5^ There is substantial in vitro, in vivo, and ex vivo evidence as well as evidence in human patients that PIPAC has superior pharmacologic properties.^6^–^9^ Because the therapeutic ratio between local and systemic drug concentration is increased by PIPAC, enhanced local efficacy together with low systemic toxicity was expected and has been demonstrated clinically.^10^ Retrospective analysis of the patient cohorts in ovarian gastric and colorectal cancer have shown encouraging results of repeated PIPAC in the palliative situation.^11^–^13^ A prospective phase 2 trial with low-dose doxorubicin and cisplatin in recurrent, platin-resistant ovarian cancer applied as a pressurized aerosol showed a clinical benefit rate of 62 % and an objective histologic regression rate of 76 %, coupled with a low incidence of severe adverse events (15 % Common Terminology Criteria for Adverse Events [CTCAE] grade 3, no CTCAE grades 4 or 5).^14^,^15^ So far, the role of PIPAC in combination with advanced cytoreductive surgery has not been determined.

We hypothesized that electrostatic precipitation may further enhance the pharmacologic properties of PIPAC as so-called electrostatic precipitation pressurized intraperitoneal aerosol chemotherapy (ePIPAC). For electrostatic precipitation, we used a commercially available, CE-certified technology developed for clearing surgical smoke from the operative field of view during laparoscopy (Ultravision, Alesi Surgical Ltd., UK). The performance and safety of Ultravision has been demonstrated in bench studies, preclinical testing, and clinical testing, including a clinical study on 30 patients undergoing laparoscopic cholecystectomy.^16^,^17^ In particular, no adverse events such as cardiac arrhythmia, modification of ECG, bowel perforations, or skin burning were reported.

The aims of this study were to assess the technical feasibility of ePIPAC, to compare the homogeneity of intraperitoneal distribution between PIPAC and ePIPAC, and to determine possible improvement of tissue uptake after ePIPAC.

## Materials and Methods

### Study Design

This was an exploratory experimental in vivo study in a large animal model comparing the effect of ePIPAC (3 animals) versus PIPAC (3 animals) versus 1 control animal (ePIPAC, no stain).

### Animal Model

The experiment was performed in compliance with the German Animal Protection Law (TierSchG 2006) and was authorized by the competent authority, State of Thuringia. ARRIVE guidelines were implemented. Seven German nonsyngeneic landrace pigs (5 females, 2 males) weighing 31.5 ± 4.5 kg were operated on by qualified surgeons under the supervision of a veterinarian. The sample size was determined in order to obtain experimental results in triplicate plus a negative control. Animals were randomly assigned to the experimental groups. Procedures were performed under general anesthesia adhering to strict protocols. The animals were euthanized under narcosis at the end of the procedure and immediately necropsied. The primary outcome measure was DT01 concentration in tissue and peritoneal fluid at the end of the procedures (quantitative measurement). Secondary outcomes were the homogeneity of blue staining within the peritoneal cavity (qualitative measurement) and the toluidine blue concentration in the peritoneal fluid at the end of the procedure (semiquantitative measurement).

### Staining Substances

DT01 are noncoding small DNA fragments designed to bait and hijack the enzyme complexes that repair DNA double-strand breaks, diverting them away from their primary objective, the double-strand breaks on chromosomes.^15^,^18^ DT01 administration with PIPAC has previously been validated.^8^ In this study, 30 mg toluidine blue and 6 mg Cy5-labeled DT01 were diluted in 1000 ml NaCl 0.9 % solution. A volume of 150 ml solution was administered via an aerosolizer (Capnopen, Capnomed GmbH, Germany) to each animal (*n* = 6). The negative control animal received 150 ml NaCl 0.9 %.

### Experimental Protocol

PIPAC was applied as described previously.^9^ After insufflation of a 12 mm Hg capnoperitoneum with a Veress needle, 2 balloon safety trocars (Kii 5 and 12 mm; Applied Medical, Germany) were inserted into the abdominal wall. The Capnopen was connected to an intravenous high-pressure injector (Arterion 7; Medrad, Germany) and inserted into the abdomen. The pressurized aerosol was applied via aerosolizer and injector. Flow rate was 30 ml/min, and maximal upstream pressure was 200 psi. The therapeutic capnoperitoneum was maintained for 30 min. The aerosol was exsufflated and the trocars removed. Identical conditions were used for the ePIPAC subjects (*n* = 3), with the additional use of the Ultravision technology. The system was activated at the point of completion of aerosol generation and the electric current was maintained for 30 min. This negative control animal received NaCl 0.9 % through ePIPAC under the above conditions.

### Electrostatic Precipitation

**Fig. 1:**
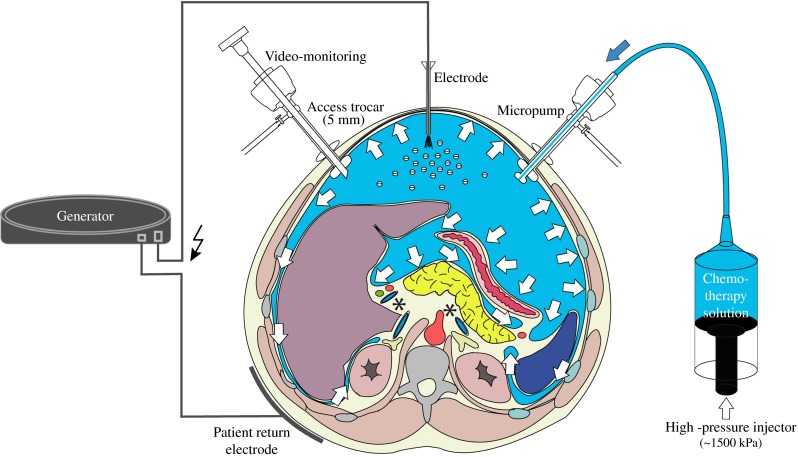
Principle of electrostatic precipitation ePIPAC. **a** Technical setting for ePIPAC, including high-pressure injector containing therapeutic solution micropump generating pressurized intraperitoneal aerosol, brush electrode for electrostatic loading of therapeutic aerosol, and return electrode (solid plate). **b** Intraoperative view of abdomen showing micropump producing aerosol and electrode actively loading this aerosol with electrostatic charges, leading to precipitation of aerosol particles

The Ultravision system integrates the following components: a generator unit (voltage 7500–9500 V, current ≤10 µA), an active cable terminating in an atraumatic stainless steel brush electrode (Ionwand) that is responsible for the electrostatic charging of aerosol particles, and a return electrode with a solid patient return plate (Fig. 1). The Ionwand emits a stream of electrons, resulting in the creation of negative gas ions. The gas ions collide with particulate matter, passing on the negative charge. The return electrode confers a weak positive charge on the subject, which results in the electrostatic attraction of the negatively charged aerosol particles to the tissue surfaces of the contained space—that is, the peritoneum.

### Analysis

Toluidine blue distribution was assessed qualitatively as described previously.^7^ Immediately after the procedure, peritoneum was sampled via biopsy; peritoneal fluid was sampled, and droplets were distributed onto filter paper to visualize the intensity of the blue stain. The tissue and peritoneal fluid samples were snap-frozen in liquid nitrogen and processed at the Institut Curie/Orsay for blinded analysis. The quantitation of DT01 in the peritoneal fluid was performed by a hybridization enzyme-linked immunosorbent assay (ELISA) using a biotin-conjugated capture oligonucleotide probe (300 μl) and a digoxigenin-conjugated detection probe (300 μl), with sequences complementary to the DT01 sequence (Exiqon, USA) in 96-well plates. Then samples were incubated with an anti-digoxigenin horseradish peroxidase–conjugated antibody (1:10,000; Roche, USA), and detection was performed by the addition of 3,3′,5,5′-tetramethylbenzidine substrate (100 μl). Absorbance was measured at 450 and 560 nm, and quantity of DT01 was calculated from calibration standards over a working range of 25–1000 ng/ml using a 4-parameter logistic curve. Because the ELISA failed to produce reliable quantification in tissues, we used fluorescent quantification, a reliable technique for assessing molecule distribution.^19^ Peritoneal tissue samples were defrosted, then crushed in phosphate-buffered saline–ethylenediaminetetraacetic acid. Fifty microliters of each extract was formed into aliquots in a 96-well plate, and fluorescence was measured with a Typhoon scanner (GE Healthcare, USA). The quantity of DT01 was calculated from a standard curve performed using peritoneal extract from untreated groups, with Cy5-labeled DT01 concentrations ranging from 0 to 1 µg/ml.

### Statistics

Size sample was determined and limited by the decision of the regulatory authority: experiments in triplicate (2 × 3), plus 1 control, for a total of 7 animals. Descriptive statistics including mean and standard deviation are provided. The null hypothesis was that DT01 concentration was equal in the peritoneal fluid and in peritoneal biopsy samples of PIPAC and ePIPAC animals. In spite of the small sample size, an exploratory comparative analysis was performed by a nonparametric test (Kruskal–Wallis test for independent samples). A *p* value of <0.05 was considered significant.

## Results

There were no technical difficulties or intraoperative complications in any of the cases. In both the PIPAC and ePIPAC groups, rapid nebulization of the toluidine blue solution within the tightly closed abdomen was observed. Videoscopic control showed immediate staining of the complete abdominal cavity in both PIPAC and ePIPAC animals, including all exposed peritoneal surfaces. Intra-abdominal organs were not mobilized.

In the ePIPAC group, electrostatic loading of the saline aerosol was technically feasible without significant aberrant conduction. The maximal allowed current intensity was not reached in the ePIPAC group, denoted by the absence of an alarm signal. After activation of the electrode in the ePIPAC group, the aerosol completely cleared from the field of view within 15 s, as documented by real-time videoendoscopy. In contrast, in the PIPAC animals, aerosol particles remained in suspension until the end of the procedure (after 30 min of steady state).

**Fig. 2:**
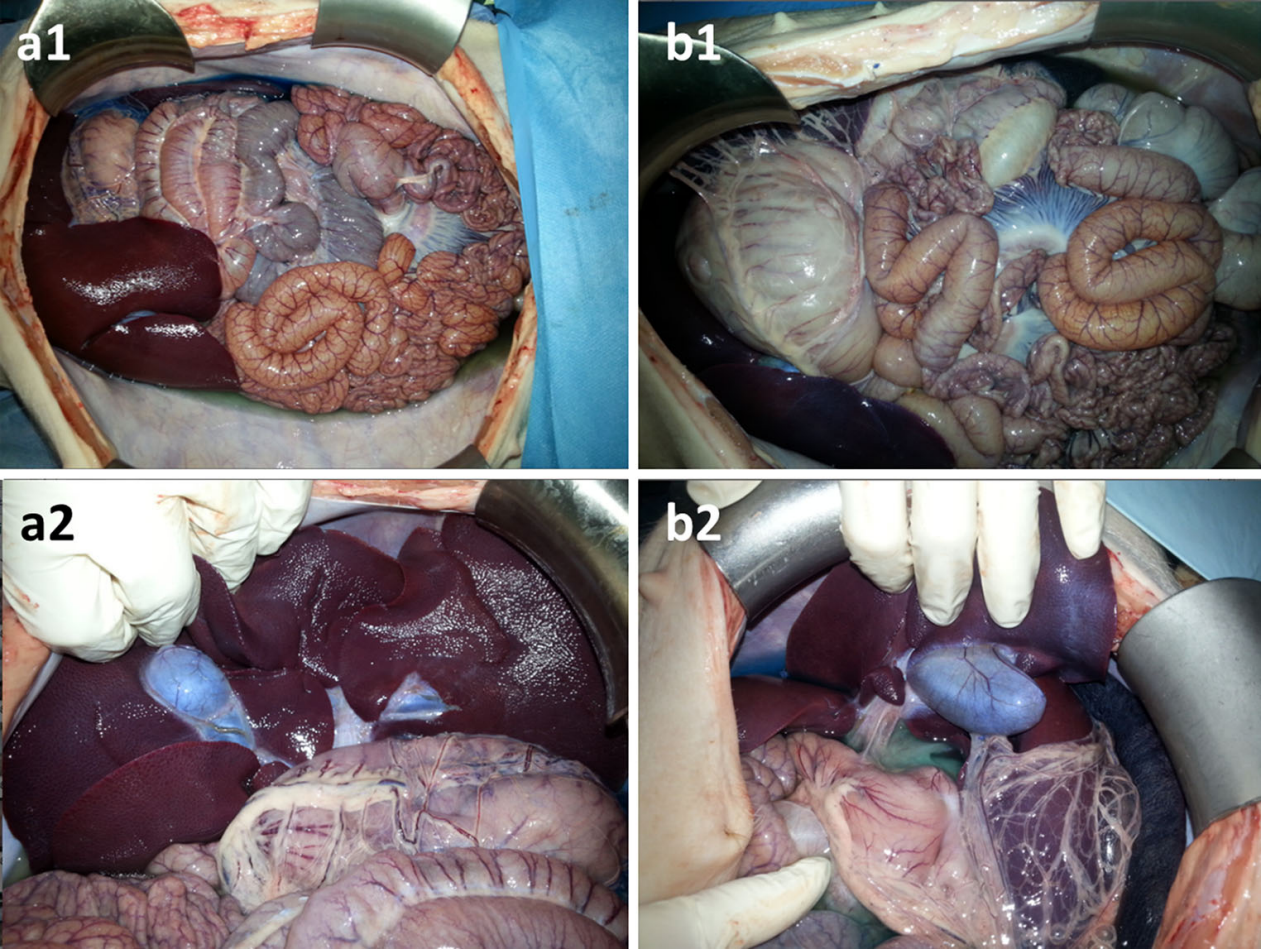
Adequacy of toluidine blue distribution. Autopsy findings in PIPAC (**a1, a2**) and ePIPAC (**b1, b2**) animals after aerosolization of low-dose toluidine blue. Staining of serosal surfaces is homogeneous in both groups. Importantly, inferior aspect of liver, including hilum and gallbladder, are stained

At necropsy, macroscopic stain distribution throughout the entire peritoneal cavity was homogeneous in the PIPAC group, including the small bowel and anterior abdominal wall and hidden surfaces such as the inferior aspect of the liver and the liver hilum. Comparable results were obtained in the ePIPAC group. In particular, no staining gradient toward or from the brush electrode was observed in the ePIPAC animals (Fig. 2). No bowel lesion or perforation was noted.

After 30 min, peritoneal fluid still demonstrated the blue staining in the PIPAC group (Supplementary material 1, arrows), whereas it was greatly reduced in the ePIPAC group. This qualitative impression was confirmed by the absence of color on filter paper with peritoneal fluid after ePIPAC. In contrast, blue staining of the peritoneal fluid was still present after PIPAC.

**Fig. 3:**
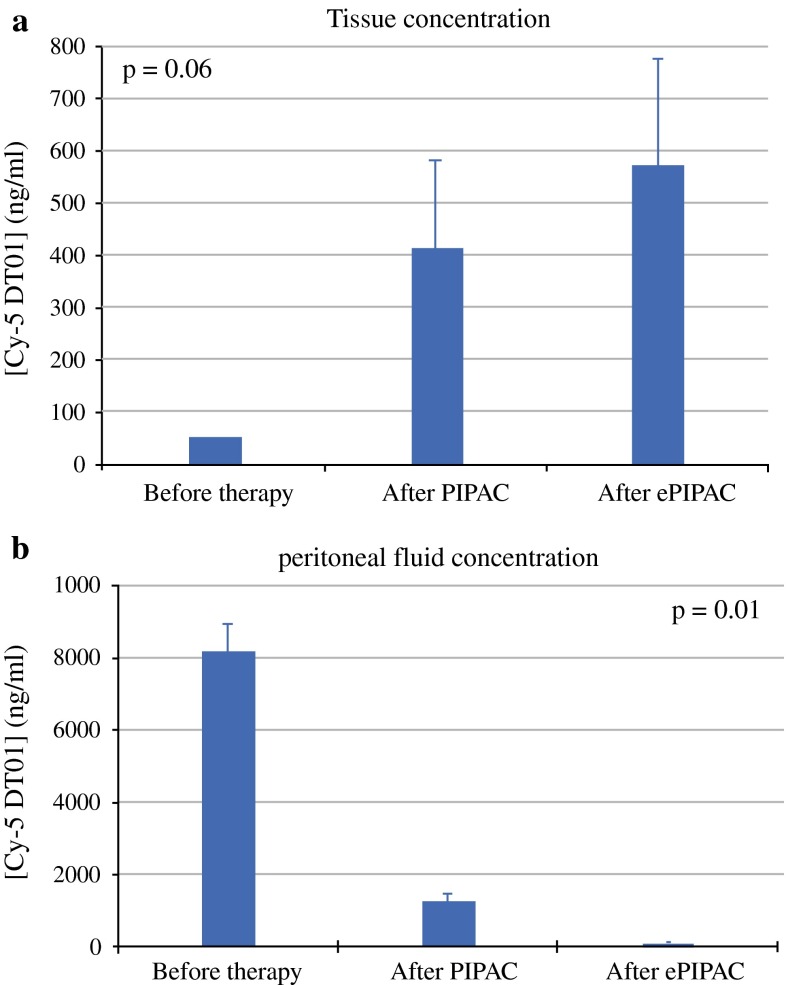
**a** Peritoneal fluid DT01 concentration showing 15 % remaining concentration after PIPAC compared to initial concentration in aerosolized solution versus 1.5 % after ePIPAC (*p* = 0.01). Whereas PIPAC allows 85 % tissue uptake, ePIPAC achieves another order of magnitude with 98.5 % absorption. **b** Tissue DT01 concentration after PIPAC vs. ePIPAC application, confirming superior uptake after ePIPAC (*p* = 0.06)

The concentration of Cy5-labeled DT01 in the peritoneal fluid was lower after ePIPAC compared to PIPAC (Fig. 3a), confirming the results obtained from the toluidine blue assessment. The mean initial DT01 concentration in the aerosolized solution was 8.93 ± 0.72 µg/ml. After PIPAC, this concentration diminished to 1.46 ± 0.21 µg/ml, indirectly documenting a tissue uptake of 83.6 % in this closed system. After ePIPAC, the concentration further dropped to 0.11 ± 0.02 µg/ml, suggesting an almost complete tissue uptake of DT01 (98.7 %). The null hypothesis could therefore be rejected (*p* = 0.01). Superior DT01 uptake after ePIPAC versus PIPAC was confirmed by tissue measurement (Fig. 3b): DT01 concentration in tissue increased from 0.05 µg/ml (background noise) before therapy to 0.41 ± 0.17 µg/ml after PIPAC application and to 0.57 ± 0.20 µg/ml after ePIPAC application (*p* = 0.06).

## Discussion

In this experimental study, we combined 3 physical principles (electrostatic precipitation, aerosol nature, and hydrostatic pressure) with the aim of further improving tissue uptake after intraperitoneal delivery, thus developing the concept of ePIPAC.

Electrostatic loading of a therapeutic aerosol was technically feasible in all ePIPAC animals in an environment highly saturated with saline solution without creating significant erratic electric currents. The aerosol was cleared much more quickly in ePIPAC animals compared to PIPAC animals. No major macroscopic differences were noted in dye distribution; in particular, there was no optical tissue staining gradient toward or from the active electrode in the ePIPAC group. At the end of the procedure, the peritoneal fluid was colorless in the ePIPAC group while the blue color was maintained in the PIPAC group, suggesting a more effective clearance of toluidine blue from the aerosol after electrostatic precipitation. Subsequent semiquantitative analysis with filter paper confirmed this clinical impression. Quantitative results obtained with the second tracer (DT01) demonstrated that transfer of the tracer from the aqueous solution to tissue surfaces was improved after ePIPAC compared to PIPAC. After PIPAC, approximately 1/10 of the DT01 was present within the peritoneal cavity, while with ePIPAC only about 1/10^2^ remained. DT01 is a much larger molecule than toluidine blue, and therefore such efficient uptake was not anticipated. This superior uptake after ePIPAC was confirmed by a higher tissue concentration than after PIPAC.

This difference in uptake represents a further improvement in the context of an earlier study in which tissue concentration of doxorubicin after PIPAC was found to be up to 200 times higher than reported after hyperthermic intraperitoneal chemotherapy (HIPEC), with only 10 % of the dose.^9^

Application of intraperitoneal chemotherapy with ePIPAC may have several potential advantages over existing techniques:

- First, if it can be shown that increased deposition efficiency translates to an increased tissue uptake, then it may increase drug uptake into tumor nodes and therefore achieve cytotoxic dose in larger nodules. This could in turn reduce the need for aggressive cytoreductive surgery and allow therapy of diffuse small bowel involvement, a contraindication for cytoreductive surgery and HIPEC.^20^
- Second, it may allow a further reduction of the dose needed to be effective. In ovarian and gastric cancer, PIPAC has been shown to be effective with 10 % of the usual systemic dose of cisplatin and doxorubicin.^12^,^14^ In colorectal cancer, PIPAC was effective with 20 % of the doses generally administered for HIPEC.^13^ Dose reduction allows not only a reduction in organ toxicity and systemic side effects but also a limit to local toxicity on the bowel and the normal peritoneum.^10^,^14^ The clinical significance of this is the possibility to use PIPAC earlier in the course of the disease as a secondary prevention of peritoneal metastasis, analogous to HIPEC, and may contribute to a lower incidence of peritoneal sclerosis.^21^–^25^
- Third, ePIPAC may allow a significant reduction of the time needed for application.
- Finally, it may simplify the occupational safety aspects of PIPAC by reducing time of potential exposure and by minimizing any residual drug evacuated at the end of the procedure.

There are several limitations to this early work. These data have been obtained in an experimental model and cannot be extrapolated to human patients without further validation. This study was not performed in an animal model of peritoneal metastasis but in healthy pigs because such a model is not available. Therefore, tracer penetration into tumor tissue could not be assessed.

It was not possible to apply toxic agents such as cytotoxic drugs within the experimental operating room as a result of the absence of high-flow ventilation. Although DT01 is a validated marker for determining tissue drug uptake, the results presented are only valid for the substances tested.^8^,^26^ It is likely that the ability to improve tissue deposition of drug substances using electrostatic precipitation will be affected by the physical characteristics of these molecules. Thus, it is not possible to extrapolate the clearance of toluidine blue and DT01 to the clearance of chemotherapy drugs. Additional pharmacologic studies are required for each drug used for ePIPAC.

## Conclusions

The therapeutic effect of ePIPAC is through a combination of aerosolization of the drug, applying a pressure across it and application of an electrostatic gradient. ePIPAC is technically feasible and improves tissue uptake of 2 tracer substances compared to PIPAC. ePIPAC has the potential to allow more efficient drug uptake, to permit further dose reduction, to significantly shorten the time required for PIPAC application, and to improve health and safety in the operating room when undertaking such procedures.

## Electronic Supplementary Material

Below is the link to the electronic supplementary material.

Supplementary material 1 (TIFF 1524 kb)
