# Supplementary figures and images for: In Vivo Feasibility of Electrostatic Precipitation as an Adjunct to Pressurized Intraperitoneal Aerosol Chemotherapy (ePIPAC)
Source: Ann Surg Oncol. 2016 Feb 2;23(Suppl 5):592–8. doi: 10.1245/s10434-016-5108-4 (PMC5149560; doi:10.1245/s10434-016-5108-4)

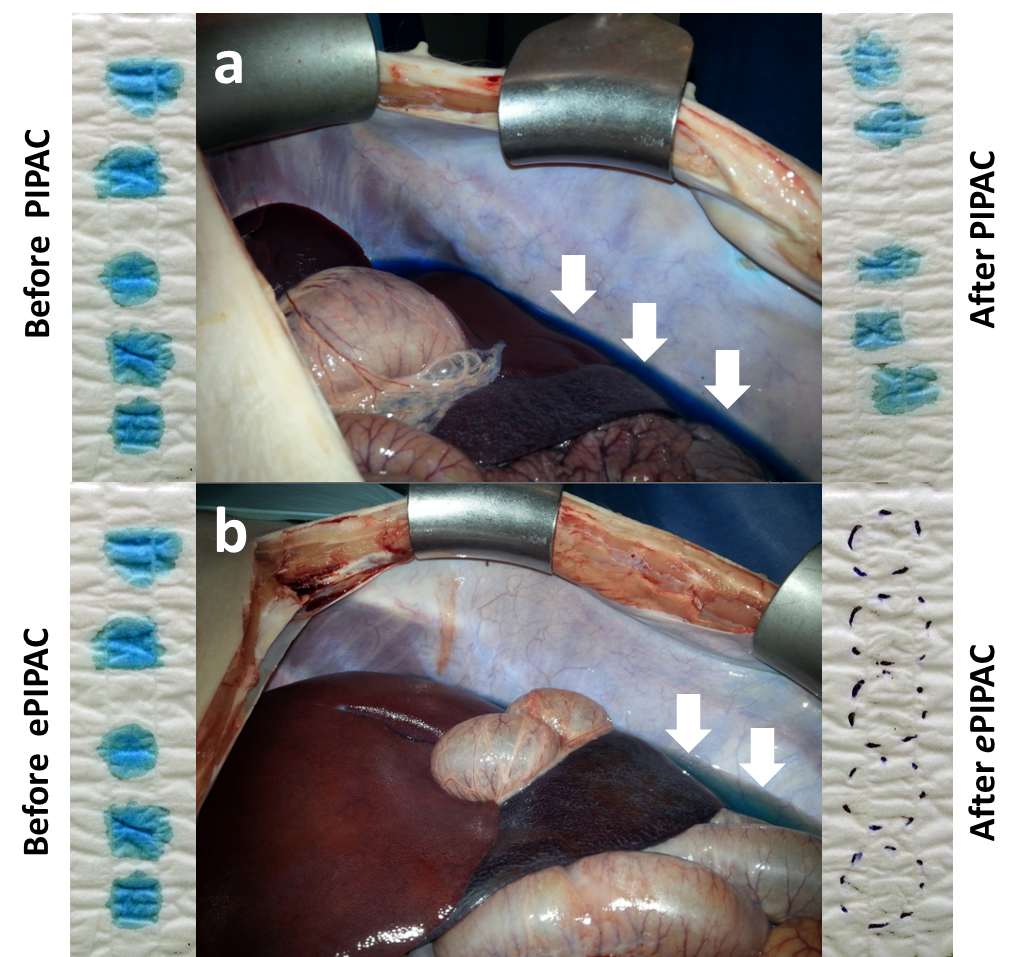

Supplement: Supplementary file 1 — Supplementary material 1 (TIFF 1524 kb) [file 10434_2016_5108_MOESM1_ESM.tif]
